# Neuropeptide Y in Alcohol Addiction and Affective Disorders

**DOI:** 10.3389/fendo.2017.00178

**Published:** 2017-07-31

**Authors:** Annika Thorsell, Aleksander A. Mathé

**Affiliations:** ^1^Center for Social and Affective Neuroscience, Department of Clinical and Experimental Medicine, Linköping University, Linköping, Sweden; ^2^Department of Clinical Neuroscience, Karolinska Institutet, Stockholm, Sweden

**Keywords:** neuropeptide Y, receptor subtypes, anxiety, stress, depression, post-traumatic stress disorder

## Abstract

Neuropeptide Y (NPY), a neuropeptide highly conserved throughout evolution, is present at high levels in the central nervous system (CNS), as well as in peripheral tissues such as the gut and cardiovascular system. The peptide exerts its effects *via* multiple receptor subtypes, all belonging to the G-protein-coupled receptor superfamily. Of these subtypes, the Y1 and the Y2 are the most thoroughly characterized, followed by the Y5 subtype. NPY and its receptors have been shown to be of importance in central regulation of events underlying, for example, affective disorders, drug/alcohol use disorders, and energy homeostasis. Furthermore, within the CNS, NPY also affects sleep regulation and circadian rhythm, memory function, tissue growth, and plasticity. The potential roles of NPY in the etiology and pathophysiology of mood and anxiety disorders, as well as alcohol use disorders, have been extensively studied. This focus was prompted by early indications for an involvement of NPY in acute responses to stress, and, later, also data pointing to a role in alterations within the CNS during chronic, or repeated, exposure to adverse events. These functions of NPY, in addition to the peptide’s regulation of disease states, suggest that modulation of the activity of the NPY system *via* receptor agonists/antagonists may be a putative treatment mechanism in affective disorders as well as alcohol use disorders. In this review, we present an overview of findings with regard to the NPY system in relation to anxiety and stress, acute as well as chronic; furthermore we discuss post-traumatic stress disorder and, in part depression. In addition, we summarize findings on alcohol use disorders and related behaviors. Finally, we briefly touch upon genetic as well as epigenetic mechanisms that may be of importance for NPY function and regulation. In conclusion, we suggest that modulation of NPY-ergic activity within the CNS, *via* ligands aimed at different receptor subtypes, may be attractive targets for treatment development for affective disorders, as well as for alcohol use disorders.

## Introduction

Neuropeptide Y (NPY), a 36 amino acid neuropeptide, was originally isolated from porcine brain using a method detecting the C-terminal amide. NPY belongs to the pancreatic polypeptide (PP) family of biologically active peptides, together with two other members, PP and peptide YY (1). The amino acid sequence for porcine NPY was determined in 1985 (2), and it was subsequently determined that the amino acid sequence is identical for species such as human, rat, porcine, and guinea pig (3). Indeed, both mRNA and peptide sequence display a high degree of conservation throughout evolution (4–6), possibly indicating preserved functional relevance.

Expression and synthesis of the PP-family of peptides is a multiple step process, also well conserved between species. The PP-family of peptides are synthesized as large protein precursors; for NPY, the 98 amino acid precursor peptide is proteolytically processed into three separate peptide products: an N-terminal signal peptide, NPY, and a 30 amino acid C-terminal flanking peptide (C-PON). The strong evolutionary conservation can be seen for both the NPY peptide and the C-PON, with the rat and human sequences showing 100 and 93% homology, respectively (7).

### Expression of NPY within the Central Nervous System (CNS)

Neuropeptide Y is predominantly expressed in cells originating from the neural crest, and it is one of the most highly expressed neuropeptides within the CNS; an expression that has been shown to be present in, but not limited to, neurons (7–9). NPY is expressed at high levels in brain regions involved in regulation of affective behavior, energy homeostasis, and memory function and plasticity. These include among others, the hypothalamus, in particular the arcuate and the paraventricular nuclei, the hippocampal formation, the amygdala, periaqueductal gray, locus coeruleus, and septum (7, 10, 11).

The amygdala is a central neurobiological substrate for mediation of stress- and anxiety-related behaviors and has strong NPY-ergic innervation. Within the amygdala, the central amygdala constitutes an output relay for the functional consequences of amygdala activation by fearful stimuli and, together with the the lateral/basolateral complex mediate anti-stress effects of NPY (12, 13).

The dorsolateral portion of the periaqueductal gray matter (PAG) has been suggested to tonically inhibit the amygdala. The PAG is involved in the behavioral output of fear responses, with subcompartments differentially involved in defensive behaviors (14). The septum is a key component in a behavioral inhibition system partaking in regulation of anxiety states. However, while important, lesions of the septum that affect anxiety-related behaviors most likely reflect effects on fibers passing through this structure, most likely belonging to hippocampal output. The dorsal hippocampus is an important component of neuronal circuitry controlling anxiety-related behaviors and stress responses and septo-hippocampal circuits are likely to be important for fear-related behaviors. Expression of NPY is high in hippocampal regions (15).

The numerous functions of NPY within the CNS, as well as its extensive expression, contribute to making the NPY system one of the most well-studied neuro-hormonal systems.

### NPY Receptor Subtypes and Function

Neuropeptide Y exerts its actions *via* four functionally relevant receptor subtypes, the Y1, Y2, Y4, and Y5 (16–19). All NPY receptors cloned belong to the superfamily of G-protein-coupled receptors but differ in their ligand affinity profiles (20–23). The Y1 receptor subtype requires the full peptide to be activated, while the Y2-subtype also can be bind C-terminal fragments of NPY. The Y4 receptor preferentially binds PP and may be referred to the pp1 receptor (17). The Y5 subtype binds similar ligands as the Y1 (24). NPY receptors couple *via* Gi/o proteins to several downstream signaling pathways, including inhibition of adenylyl cyclase, activation of mitogen-activated protein kinase, regulation of intracellular calcium (Ca2+) concentrations, and activation of G-protein-coupled, inwardly rectifying potassium (K+) channels (25, 26).

The predominantly postsynaptic Y1 receptor requires the intact NPY sequence for recognition and activation and is the subtype mediating antianxiety and antidepressant actions of NPY (13). Activation of the Y1 receptor decreases levels of experimental anxiety, alleviates post-traumatic stress disorder (PTSD) and depression-like behavior, predominantly *via* actions in the amygdala and hippocampus (13, 27–29). The presynaptic Y2 receptor is, in addition to intact NPY, also activated by C-terminal fragments of NPY, such as NPY 13–36 and NPY 3–36 (20). The Y2 subtype functions as a heteroreceptor, affecting presynaptic release of NPY and classical neurotransmitters, including GABA and glutamate, as well as norepinephrine (20, 30, 31). The Y4 receptor has low affinity for NPY and is primarily the target for PP, which, as mentioned also, is a member of the PP-family of peptides (32). The Y5 receptor was initially thought to be the exclusive receptor regulating NPYs effect on feeding behavior (33); however, the orexigenic effects of NPY have since been determined to also involve the Y1 and the Y2 receptor subtypes (34–36).

Within the mammalian CNS, NPY receptor subtypes are expressed in regions overlapping with NPY expression and involved in regulation of anxiety and stress, depression, energy homeostasis, and memory function. These regions include the previously mentioned amygdala, hypothalamus, and hippocampus, and also the periaqueductal gray (37), septum (38), and the locus coeruleus (39).

## Stress and Anxiety

### Early Findings

An early finding for CNS action of NPY was a long-lasting synchronization of the EEG pattern (40). This is similar to the effects of sedative/anxiolytic compounds such as bensodiazepines or barbiturates. Furthermore, i.c.v. administration of central NPY suppressed baseline as well as novelty-induced locomotor activity (41). Another early finding, the prevention of formation of gastric erosions, also indicated a role of NPY in regulation of stress-related events and, possibly, anxiety-related behavior (42). Early on, anxiolytic-like effects of NPY were demonstrated using the elevated plus-maze (EMP), the social interaction test, as well as “conflict tests” such as the Geller–Seifter and the Vogel punished drinking conflict test (43, 44). Here, spatial or social exploration is suppressed by fear of open spaces, and unfamiliar conspecifics, respectively, and restored by bensodiazepines, and also NPY (29, 43, 45). In the light–dark compartment test, a model conceptually related to the elevated plus-maze similar findings was reported (46). Finally, in the fear-potentiated startle model, which is based on fear potentiation rather than inhibition of behavior, NPY effectively reverses the potentiation of the acoustic startle response, which occurs upon presentation of a conditioned fear stimulus, but does not affect basal, unconditioned startle (45).

### Stress and Homeostasis

Responses that may be beneficial in an acute situation may become adverse under repeated circumstances. This is in particular true for stress responses, which when chronically activated may cause damage and become the basis for or accelerate disease conditions (47). Stress as a term refers to evolutionary highly conserved processes involving perception, appraisal, and response to threatening, challenging, and/or possibly harmful stimuli. Homeostasis refers to consistency of internal parameters within a normal range, while allostasis is the process of reestablishing homeostasis beyond the normal range that entails changing the homeostasis to match external demands in response to a challenge. Allostasis links the brain with the endocrine and immune systems to coordinate appropriate responses to a stressor (48, 49).

In addition to being intrinsically harmful, (chronic) stress and accompanying alterations in homeostatic balance have also been shown to increase vulnerability to addiction (50–52). Drug intake and withdrawal also in themselves act as stressors leading to a disruption of the homeostatic state and constitute a mechanism underlying progression from drug/alcohol use to abuse (53, 54). Furthermore, repeated exposure to and withdrawal from drug use leads to increased sensitivity to stress and an increased behavioral stress response (55). A hallmark of addiction is the risk for relapse following a period of abstinence. During progression from drug use to abuse, alterations in hypothalamic as well as extra-hypothalamic structures, such as the amygdala, lead to an increased stress sensitivity (51, 55–59). Stress-induced relapse is a model frequently used in preclinical settings and involves exposure to a stressor (for example, yohimbine or foot shock (60–63), for example) which then leads to the reinstatement of a previously extinguished behavior, i.e., drug taking.

Affective disorders including anxiety and depression affect as many as 1 in 4 individuals during their lifetime, and are, together with alcohol use disorders, major causes of “Years of life lived with disability” in all ages and “Years of life lost because of premature death” as a consequence of illness itself and due to depression comorbidity with, e.g., cardiovascular disease and a high suicide rate (64–69). Depression is more prevalent in women, while alcohol use disorders are more prevalent in men (65, 70). The frequency and prevalence of these disorders are increasing due to demographic changes (longer life expectancy) and, possibly, due to improved diagnostic procedures.

Currently available pharmacological treatments have limited efficacy, about one-third of patients do not respond or are only partial responders. Thus, there is a major unmet medical need, and neuropeptide systems may offer opportunities to develop novel treatments to alleviate it.

### NPY in Stress and Anxiety-Related Behavior

As previously mentioned, central administration of NPY was early on shown to mimic anxiolytic and sedative effects of compounds such as barbiturates and benzodiazepines. Later studies, using both rats and mice, have confirmed and extended these initial findings to an extensive range of experimental models, including conflict tests, fear-potentiated startle, and different mazes (43–45, 71). Consistent with effects of NPY administration, overexpression of NPY was shown to exert anxiolytic effects. Using an NPY transgenic rat model with hippocampal NPY overexpression (72), it was demonstrated that increased hippocampal NPY activity led to a behavioral insensitivity to restraint stress on the elevated plus maze, absent fear suppression of behavior in a punished drinking test, and impaired spatial learning in the Morris water maze. Additionally, localized overexpression of NPY within the amygdala led to decreased anxiety, as well as alcohol intake, in rats, further confirming a role for endogenous NPY in regulation of anxiety-related behavior (73). This was also confirmed by the finding that rats with an innate higher number of NPY-positive cells in the central amygdala displayed less anxiety-like behavior in the light–dark box model (74). However, while the amygdala has long been known to regulate fear and anxiety-related behavior, as well as being considered a site of storage of fear memories, newer findings suggest that the prefrontal cortex (PFC) is essential in the regulation of amygdala-dependent memories and fear expression (75). Dysregulation of fear related memories are of especial importance in patients with PTSD. Within the PFC, activation of the prelimbic cortex (PrL) enhances the expression of fear, while an elevated activity in the infralimbic cortex (IL) enhances fear extinction. It was recently shown that the pyramidal neurons in the PrL receive a direct inhibitory input, which is mediated by bipolar NPY(+)-GABAergic projection neurons in the IL (76). Additionally, infusion of NPY into the IL impairs retrieval of fear extinction without affecting depression-like behavior or working memory (77). Further, NPY is markedly reduced in several brain regions in a well-defined rat model of PTSD, exposure to predator scent (78–80) and, crucially, direct NPY administration into the CNS or intranasally administered NPY counteracts PTSD symptoms (81–85). Amygdala–PFC connections have indeed been demonstrated to be of great clinical relevance in PTSD (86). Interestingly, changes in the Npy gene (rs16147 T>C polymorphism) represent a risk factor for expression of negative affect in individuals exposed to adversity in early childhood (87).

The Y1 receptor subtype has been shown to mediate the anxiolytic effect of NPY within the amygdala (13, 88), with the presence of the receptor being required for this effect (27). Additionally, administration of Y1 receptor antagonists into the lateral ventricles or the basolateral nucleus of the amygdala induced anxiogenic effects in rats (89, 90). In contrast, activation of the Y2 receptor subtypes is anxiogenic, following ventricular administration or local injection into the basolateral amygdala (91, 92). This is consistent with the proposed localization of Y2 receptors presynaptically (93) and has been suggested to be due to a self-regulatory mechanism where activation of the Y2 receptor leads to decreased release of NPY (and regulation of GABA/glutamate dependent on neurobiological substrate). In line with this, blockade of Y2 receptors could be expected to be anxiolytic. Indeed, using different antagonists aimed at the Y2 receptor anxiolytic-like effects have been demonstrated in animal models, including the elevated plus-maze and conflict tests (94–96). Additionally, Y2 receptor knockout showed a low-anxiety phenotype in the elevated plus maze and open field tests, suggesting that in addition to limiting NPY-release, the Y2 receptor may counterbalance anxiolytic effects of NPY (95, 96). Deletion of Y2 receptors also lead to reduced neuronal activation in brain areas of interest following stress exposure (97). Furthermore, brain region-specific deletions of the Y2 receptor gene within the basolateral and central amygdala generated an anxiolytic phenotype (98). On the contrary, administration of NPY (13–36), a Y2 receptor specific agonist, into the vicinity of the locus coeruleus produced anxiolytic effects (39), indicating regional differences in effects of Y2 receptor activation.

Although Y4-knockout mice display reduced anxiety-related and antidepressant activity, as well as enhanced locomotor activity in behavioral tests (99, 100), direct activation of the Y4 receptor did not show any effect on anxiety-related behavior (101). A putative interaction between Y2 and Y4 receptor subtypes was suggested by an amplified anxiolytic-like effect of a double Y2/Y4 knockout (102). However, further elucidation of the involvement of the Y4 receptor subtype is hampered by the lack of specific ligands aimed at the receptor. The Y5 subtype has also been implicated in regulation of affective behavior, but it is difficult to determine the contribution of this receptor subtype due to its close relationship with the Y1 subtype. Conflicting results exist as to whether Y5 antagonists may reduce food intake and have anxiolytic or antidepressant-like effects (103, 104). In an animal model, the Y1 and Y5 receptors were shown to have overlapping functions as well as expression in regions regulating anxiety. Conditional removal of the Y1 receptor in Y5 receptor-expressing neurons in juvenile mice leads to higher anxiety but no changes in hypothalamus–pituitary–adrenocortical axis activity, under basal conditions or after acute restraint stress (105).

## NPY as an Antidepressant

The relationship between anxiety and depression is that of overlapping conditions. Symptoms of anxiety and depression commonly co-exist, and both disorders are thought to reflect maladaptive changes in stress-responsive systems (106).

In depression, there have been reports on reductions in gray matter volume and glial density in regions mediating the cognitive aspects of depression, i.e., the PFC and the hippocampus (107). In addition, functional studies show that activity within the amygdala and subgenual cingulate cortex is chronically increased in depressed individuals while reverting back to normal levels with successful treatment (108, 109). In rodents, exposure to chronic mild stress, a model used to induce a depressive-like state, increased activity, measured as c-Fos response, within the amygdala, medial habenula, and IL in rats susceptible to the stress effects (110). Within the hippocampus, NPY modulates synaptic activity and inhibits hippocampal excitability, having distinct effects on memory function (30, 111).

Indeed, central administration of NPY was shown to exert antidepressant-like effects in the forced swim test (FST), indicated by a dose-dependent increase in swimming and a decreased immobility (112). In another study, intracerebroventricular administration of NPY in olfactory bulbectomized rats, a rodent model of depressive-like symptoms, resulted in attenuation of increased behavioral irritability (113), indicating a possible therapeutic role of NPY in reducing depression-like behaviors. NPY has also been shown to reverse tricyclic antidepressant treatment-resistant depression induced by central administration of adrenocorticotropic hormone (114). Furthermore, NPY has been shown to modulate effects of antidepressant treatments such as imipramine and for exploratory treatments such as agmantine and other neuropeptides (115–117).

With regard to receptor subtypes mediating the antidepressant effects of NPY, activation of the Y1 receptor subtype has been shown to have direct antidepressant-like effects, as well as to modulate effects of antidepressant treatment (28, 115). Recently, intranasal administration of both NPY as well as a peptidergic Y1-agonist to rats was shown to have antidepressant-like effects (82, 83, 118) Furthermore, it was recently shown that chronic treatment with a Y5 receptor antagonist produced antidepressant-like effects in the rat chronic mild stress model and reversed depressive-like behavioral changes in the FST and prevented degeneration of astrocytes in the mPFC (104, 119).

Reduced NPY expression, both mRNA and protein, may constitute a risk for depression and anxiety-related behaviors. In a study of Fawn Hooded rats, an animal model of depression, decreased NPY concentrations were found in hippocampus compared to control animals (120). In another genetic animal model of depression, the Flinders Sensitive Line (FSL) rats, decreased NPY protein was found in the hippocampal CA region, while Y1 binding sites were increased. On the other hand, NPY was increased in the arcuate nucleus of the hypothalamus, compared to the non-depressed control Flinders Resistant Line (FRL) rats (121–123). For gene expression, Y1 receptor mRNA was decreased in several cortical and limbic regions in FSL rats compared with FRL rats (122). Considering the increased prevalence of depression with age, the observation that cell loss of the NPY-positive cells in the dentate gyrus is enhanced in the depressed FSL animals as they age compared to the FRL line confirm their use as a depression model (124). Consistent with these findings, decreased NPY in selected brain regions has been found in several models of dysregulated emotionality and stress, such as learned helplessness (125), maternal separation/deprivation (126, 127), chronic mild stress (128), social isolation (129), PTSD (79), and acute as well as early-life stress (130, 131), as well as in animal models of alcohol use such as an alcohol-preferring rat strain (132).

## Modulation of NPY Expression and Activity by Stress or Treatment

Exposure to stress or fearful stimuli, or treatment with anxiolytic or antidepressant drugs, affects CNS expression and function of NPY, the relationship being bidirectional. Acute stress significantly decreased NPY expression within the amygdala, an effect accompanied by anxiogenic behavior as measured on the elevated plus-maze (130). The reverse relationship was found when the stressor was applied repeatedly, indicating an innate mechanism for adaptations in amygdala NPY dependent on context or exposure frequency (133).

With regard to treatments, antidepressants administered orally to rats increased NPY in frontal cortical regions and the hypothalamus, as did electroconvulsive (ECS) treatment (134, 135). Lithium treatment was shown to increase NPY protein and mRNA levels in several brain regions in rats, such as the striatum, hippocampus, frontal and occipital cortices, and the entorhinal cortex (136–139). Early studies of ECS demonstrated that hippocampal and cortical NPY levels increased after repeated ECS in rats (140–142). Treatment of experimental animals with SSRIs has yielded differential effects on NPY levels in different brain regions. Interestingly, antidepressant effects of exercise may be related to alterations in hippocampal NPY levels (143, 144).

## NPY in Alcohol Use Disorders

Substance use disorders correlate significantly with prevalence of mood and anxiety disorders that develop independent of intoxication and withdrawal (145–147). Specifically, overconsumption of alcohol is commonly associated with anxiety and depression (148). Animal models have shown that acute ethanol administration produces dose-dependent anxiolytic effects short term (149), while acute high doses produce withdrawal-induced anxiety (150). Additionally, greater alcohol intake has been associated with states of anxiety (151). The highly prevalent comorbidity has generated interest in anxiolytic and antidepressant drugs as putative treatment targets in alcohol use disorders (152).

Accumulating evidence points to a key role of NPY in the modulation of the development of alcohol dependence. Alcohol consumption is increased in mice with a null mutation of the NPY gene, but decreased in transgenic NPY overexpressing subjects (153). Furthermore, differences either in electrophysiological responses to exogenous NPY or in peptide concentrations in specific brain regions have been found in rat strains selected for high and low alcohol preference (132, 154, 155). Further support for an involvement of NPY and its receptors in the behavioral consequences of alcohol dependence come from animals with a history of alcohol dependence induced *via* alcohol vapor exposure. Here, changes in NPY-like immunoreactivity (156), stress-responsivity (55), and brain activation patterns (157) were seen. These findings indicate that alterations in NPY-related systems may underlie some of the behavioral changes induced by a history of alcohol vapor exposure, and suggesting that NPY, or analogs thereof, may modulate alcohol-induced behavioral modifications. Alterations in NPY system expression and function are seen for many drugs of abuse (158).

Administration of NPY into the ventricles reduced alcohol intake in alcohol-preferring P rats (159), as well as in vapor-exposed animals (160), while this effect was absent in animals without a history of dependence or the appropriate genetic background (161). Reduction of alcohol intake following NPY infusion in predisposed animals might relate to its anxiolytic effect, since alcohol dependence is accompanied by an increased sensitivity to stress (55). This relates to the clinical context, in that, clinical studies have shown a correlation between anxiety levels and subsequent alcohol abuse (152).

The amygdala is a central neurobiological substrate in stress- and anxiety-related behavior, as well as in modulation of alcohol intake (162, 163). Amygdala lesions disrupt anxiety-related behavior and reduce alcohol consumption (164, 165). Some lines of alcohol-preferring rats also exhibit higher anxiety-like behaviors and lower amygdala NPY levels (166). However, as an illustration of the complexity of alcohol use disorders, increased alcohol intake due to selective breeding can also be accompanied by reduced anxiety-like behavior in rodents (166). An infusion of NPY into the central nucleus of the amygdala in alcohol-preferring rats normalizes both anxiety behaviors (assessed using the light/dark box exploration test) and alcohol intake (167). Conversely, direct injection of NPY into the paraventricular nucleus of the hypothalamus actually increases alcohol consumption (168), an effect that may illustrate the caloric content of ethanol. Additionally, elevated NPY signaling in the nucleus accumbens and/or striatum may contribute to the increased sensitivity to ethanol-induced behavioral sensitization. Reduced expression of ethanol-induced behavioral sensitization was seen following activation of Y2 receptors in the nucleus accumbens (169).

Alcohol use disorders are characterized by, among other things, escalated consumption over time, an inability to stop intake despite adverse consequences, and relapse to alcohol taking following periods of abstinence. Periods of consumption are interspersed with periods of alcohol withdrawal and abstinence. Alcohol withdrawal induces acute anxiety (170), which can be alleviated by known anxiolytics. Alcohol withdrawal affects NPY expression, and withdrawal-induced decreases of NPY within the central amygdala likely contribute to increased GABAergic tone in alcohol-dependent animals. It has been shown that application of exogenous NPY normalizes dependence-induced increases in GABA release in CeA (171).

### NPY Receptor Function in Alcohol Use Disorder

Neuropeptide Y infusion into the CNS reduces alcohol intake in animal models of escalated intake, and an overexpression of NPY within the amygdala reduces alcohol intake in a choice model (72, 73). The direct effect of NPY in reducing alcohol intake is most likely due to an increased activation of the Y1 receptor subtype. It was recently demonstrated that Y1 receptor activation in the bed nucleus of the stria terminalis suppressed binge alcohol drinking and that the underlying mechanism was an enhanced inhibitory synaptic transmission specifically in CRF neurons *via* a Gi-mediated, PKA-dependent postsynaptic mechanism (163). Furthermore, central infusion of NPY, a Y1 receptor agonist, and a Y2 receptor antagonist significantly blunted binge-like alcohol drinking in C57BL/6J. In that study, binge-like alcohol drinking reduced NPY and Y1 immunoreactivity in the central nucleus of the amygdala, while 24 h of alcohol abstinence after a history of binge-like drinking promoted increases of Y1 and Y2R expression. The binge-like alcohol drinking augmented the ability of NPY to inhibit GABA (172).

Some data indicate that Y2 receptor antagonism proposedly leads to increased NPY in the synaptic cleft, thereby functioning as an indirect Y1 receptor agonist. Indeed, results from Y2 knockout mice in models of alcohol intake (173), as well as in anxiety and depression models (95), indicate that antagonism of the Y2 receptor may modify these behaviors. Furthermore, central administration of the Y2 receptor antagonist BIIE0246 suppressed self-administration of a sweetened alcohol solution in rats, and post-dependent animals showed increased sensitivity to this effect (174, 175). However, using a different small molecule, non-peptidergic antagonist, JNJ-31020028 (176), in high alcohol-preferring rats as well as outbred Wistar rats, no effect on alcohol intake-related behaviors or relapse to alcohol seeking could be detected (177). The differential effects may be due to structural differences in the used ligands.

With regard to the Y5 receptor subtype, NPY activity at this receptor subtype can modulate ethanol reinforcement in mice (178). Furthermore, in a high alcohol-preferring rat line, antagonism at the Y5 receptor can reduce alcohol intake (179).

In addition to increased alcohol intake, a hallmark of addiction is the risk to relapse following periods of prolonged abstinence (52–54). The potential role of NPY as well as NPY receptor ligands in preventing relapse of alcohol intake in dependent animals has been explored in several experiments. Thus, NPY-administered ICV blocked reinstatement of alcohol seeking induced by the pharmacological stressor yohimbine, an alpha-2 adrenoreceptor antagonist (161).

## Clinical Studies

Decreased levels of CSF NPY have been found in patients with affective disorders, patients who had a history of suicide attempt, PTSD, and dementia (180–185). PTSD patients have lower plasma and CSF NPY levels than healthy controls (186, 187). Challenge studies have also demonstrated differences between in PTSD patients and controls. In healthy subjects, intravenous administration of yohimbine has been reported to induce anxiety as well as relapse to alcohol seeking and craving for alcohol, increased plasma NPY, highlighting the role of NPY in regulating anxiety (187, 188); this effect was attenuated in PTSD. NPY levels were also positively correlated with cortisol levels and behavioral performance under stress (189–191). The reduced levels of NPY in CSF have been shown to be accompanied with reductions in NPY immunoreactivity and mRNA in postmortem brain tissue (192). In a study with patients with depression and anxiety, serum NPY levels were lower in the patients than in the controls. Serum NPY levels were increased by treatment with escitalopram and venlafaxine in the patients with depression, but not in the patients with anxiety (193). Regulation of NPY levels in circulation is regulated in part by the enzyme dipeptidylpeptidase 4 (DPP4). DPP4 has been shown to have lower activity in depressed patients, an effect reversed by antidepressive treatment (194, 195).

## Genetics and Epigenetics of NPY

Affective disorders as well as alcohol use disorders have strong genetic contributions (196, 197). In an early study of the genetics of alcohol dependence, linkage analysis on the F2 intercross progenies of P and non-preferring rats revealed a chromosomal region containing a NPY precursor gene (198, 199). Within the Npy gene, a number of functional single-nucleotide polymorphisms (SNPs) exist within the Npy gene. NPY haplotypes were found to predict levels of NPY mRNA in postmortem brain and levels of plasma NPY, as well as emotion-induced activation of the amygdala (200). A SNP (rs16147) located in the promoter region alters NPY expression *in vitro* and seems to account for more than half of the variation in expression *in vivo* (87). In depression, reductions in NPY levels are associated with a preproNPY SNP (201, 202). The rs16147 SNP was associated with impaired antidepressant treatment response in patients with anxious depression (203, 204), and low-expression NPY genotypes were also found to be overrepresented in subjects with major depression (205).

Within the Npy gene, a thymidine(1128)-to-cytosine(1128) polymorphism (T1128C; rs16139), which results in a substitution of Leu(7) by Pro(7) in the signal peptide part of pre-pro-NPY, was identified in relation to serum cholesterol (206). The minor allele of the Leu7Pro polymorphism in the NPY gene has been associated with higher processing into mature NPY and higher CSF NPY levels (201). The cytosine 1128 (Pro7) allele was shown to be rare in a depression population and has been suggested to play a protective role against depression (202, 207). Based on animal literature, the Pro7 allele was suggested to be associated with elevated alcohol consumption in humans. Indeed, the frequency of Pro7 allele was higher in European Americans subjects with alcohol dependence compared with healthy controls. These findings suggest that in humans, the Pro7 allele of leu7pro may be a genetic vulnerability for pathological alcohol consumption and dependence. Conflicting data exist with regard to the contribution of the Pro7 allele in alcohol dependence (208–210). Other NPY gene polymorphisms have been associated with alcohol dependence including a polymorphism at the 602 position in the 5′ region and a C to T substitution at the 5671 position (211). Additionally, in rhesus macaques, it was suggested that a polymorphism within the Npy gene promoter may be associated with susceptibility to alcohol use disorders (212). Furthermore, the increased drinking of P rats may be related to NPY-ergic activity in this selectively bred rat line (213–215).

Neuropeptide Y Y5 receptor variants have also been found to contribute to the etiology of panic disorder in a population of German patients, supporting the evidence for a risk locus on chromosome 4q31–q34 in anxiety disorders (216). With regard to the Y1 and Y2 receptor subtypes, limited data on SNPs are available. For the Y2, haplotypes containing a SNP within the first intron (rs17376826 SNP) have strong associations with body mass index, but relations to stress, anxiety, or alcohol addiction have not been examined (217). Additional Y2 polymorphisms, rs4425326 and rs6857715, have been associated with severe alcohol dependence, comorbid alcohol and cocaine, and cocaine dependence in European American population (218). Furthermore, the prevalence of current smokers was greater among Japanese men having the rs4425326 C-allele compared to ex-smokers (219).

Epigenetic mechanisms have so far only to a limited extent been shown to be involved in stress- and nutritional-regulation of NPY expression and function. Perinatal malnutrition has stress-like effects on offspring in animal models (220–224) and has been shown to alter DNA methylation of CpG dinucleotides in the proximal promoter region of the NPY gene within the hypothalamus at 16 and 100 days of age, compared to control rats (225). Additionally, rearing of newborn rats on a high-carbohydrate diet, shown to induce hyperinsulinemia, increases acetylation of lysine 9 in histone 3 (H3K9) for the NPY gene, without changes in histone methylation (H3K9). These findings were consistent with the changes in the expression levels of NPY, suggesting that epigenetic mechanisms regulate NPY levels in response to nutritional stress, at least within the hypothalamus. Within the amygdala, NPY protein levels were shown to be decreased in the CeA and MeA of rats with access to intermittent alcohol during adolescence (226). The authors additionally showed that histone H3K9/14 acetylation was decreased in the Npy promoter in the amygdala of alcohol-exposed adult rats compared to controls.

In the context of stress and affective disorders, the epigenetic contribution to regulation of NPY expression and function needs to be further elucidated.

The SNP in the rat NPY gene promoter (C/T; rs105431668) affects *in vitro* transcription and DNA-protein interactions. In a rat model of depression, the FSL-line, and its counterpart, the FRL line, the presence of the C-allele enables binding of a transcription factor (CREB2) and a histone acetyltransferase (Ep300). It was determined that the C-allele is only present in the FRL rat line and that its presence correlates with increased hippocampal levels of NPY mRNA and H3K18 acetylation, a gene-activating histone modification maintained by Ep300 (227, 228). This finding illustrates a direct epigenetic mechanism for regulation of NPY expression and function. At the very least, the finding opens up an interesting avenue of exploration for genetic/epigenetic interactions in affective disorders. Furthermore, this suggests that different populations due to their genetics may be differentially susceptible to exposure to stressful, adverse events both during development and in adulthood.

As far as other epigenetic mechanisms, histone acetylation has been shown to affect anxiety-related behavior as well as NPY expression within the amygdala. Specifically, more pronounced deficits in histone acetylation were suggested to be involved in lower NPY expression in the amygdala of P rats, and, thereby, operative in controlling anxiety-like and alcohol-drinking behavior (229, 230). With regard to small RNAs, here microRNA (miRNA) and NPY, a study indicated that deletion of Dicer, an enzyme cleaving pre-miRNAs into miRNAs, in mice leads to decreased expression of NPY mRNA within the hypothalamus (231). However, the authors indicated that this may be a compensatory mechanism due to the genetic modification, and not a direct cause–effect relationship. Thus, miRNA involvement in NPY gene expression remains to be elucidated.

## Concluding Remarks

Affective disorders, including anxiety and PTSD, and alcohol use disorders are major causes of “Years of life lived with disability” in all ages and “years of life lost because of premature death.”

Pathophysiologies are insufficiently understood, and currently available drugs in the clinic are only partially effective. While dysregulation of the monoaminergic systems may be a sufficient cause, there is ample evidence that dysregulation of the glutamatergic signaling and changes in neuropeptides, in particular NPY may result in same phenotypes. Consequently, there is an urgent unmet medical need to develop novel treatments that would focus on those targets. Above review of NPY illustrates its important role in physiology as well as pathophysiology of several brain disorders with dysregulated emotionality and points to its potential as a therapeutic agent that can be administered intranasally.

## Author Contributions

Both AT and AM particpated in drafting, writing, and editing the manuscript.

## Conflict of Interest Statement

The authors declare that the research was conducted in the absence of any commercial or financial relationships that could be construed as a potential conflict of interest.
